# Inflammatory Biomarkers in Atherosclerosis: Pentraxin 3 Can Become a Novel Marker of Plaque Vulnerability

**DOI:** 10.1371/journal.pone.0100045

**Published:** 2014-06-17

**Authors:** Akihiro Shindo, Hiroshi Tanemura, Kenichiro Yata, Kazuhide Hamada, Masunari Shibata, Yasuyuki Umeda, Fumio Asakura, Naoki Toma, Hiroshi Sakaida, Takao Fujisawa, Waro Taki, Hidekazu Tomimoto

**Affiliations:** 1 Department of Neurology, Mie University Graduate School of Medicine, 2–174 Edobasih, Tsu, Mie, Japan; 2 Department of Neurosurgery, Mie University Graduate School of Medicine, 2–174 Edobashi, Tsu, Mie, Japan; 3 Institute for Clinical Research, Mie National Hospital, 357 Ozatokubota, Tsu, Mie, Japan; Università degli Studi di Milano, Italy

## Abstract

Inflammation is crucially involved in the development of carotid plaques. We examined the relationship between plaque vulnerability and inflammatory biomarkers using intraoperative blood and tissue specimens. We examined 58 patients with carotid stenosis. Following carotid plaque magnetic resonance imaging, 41 patients underwent carotid artery stenting (CAS) and 17 underwent carotid endarterectomy (CEA). Blood samples were obtained from the femoral artery (systemic) and common carotid artery immediately before and after CAS (local). Seventeen resected CEA tissue samples were embedded in paraffin, and histopathological and immunohistochemical analyses for IL-6, IL-10, E-selectin, adiponectin, and pentraxin 3 (PTX3) were performed. Serum levels of IL-6, IL-1β, IL-10, TNFα, E-selectin, VCAM-1, adiponectin, hs-CRP, and PTX3 were measured by multiplex bead array system and ELISA. CAS-treated patients were classified as stable plaques (n = 21) and vulnerable plaques (n = 20). The vulnerable group showed upregulation of the proinflammatory cytokines (IL-6 and TNFα), endothelial activation markers (E-selectin and VCAM-1), and inflammation markers (hs-CRP and PTX3) and downregulation of the anti-inflammatory markers (adiponectin and IL-10). PTX3 levels in both systemic and intracarotid samples before and after CAS were higher in the vulnerable group than in the stable group. Immunohistochemical analysis demonstrated that IL-6 was localized to inflammatory cells in the vulnerable plaques, and PTX3 was observed in the endothelial and perivascular cells. Our findings reveal that carotid plaque vulnerability is modulated by the upregulation and downregulation of proinflammatory and anti-inflammatory factors, respectively. PTX3 may thus be a potential predictive marker of plaque vulnerability.

## Introduction

Atherosclerosis is a systemic arterial disease involving the intima of large- and medium-sized systemic arteries, including the aorta, carotid, coronary, and peripheral arteries. Currently, atherosclerosis is assumed to result from complex endothelial dysfunction induced by elevated and modified low-density lipoproteins, hypertension, smoking-induced toxins, free radicals, pathogenic microorganisms, shear stress, and/or a combination of these and other factors that lead to a compensatory inflammatory response [1]. Inflammation is currently recognized as an important factor involved in the development, progression, and rupture of atherosclerotic plaques, and subsequently, thrombosis [2]–[6]. Moreover, endothelial dysfunction is characterized by decreased nitric oxide synthesis and local oxidation of circulating lipoproteins and their entry into the vessel wall [7].

The levels of circulating inflammatory biomarkers, including high-sensitivity C-reactive protein (hs-CRP), and interleukin (IL)-6, are important predictors of future vascular events [8], [9]. Recently, pentraxin 3 (PTX3) has also been implicated in cardiovascular events [10].

PTX3, a prototypical member of the long pentraxin family, has a C-terminal sequence homology with the classic short pentraxins, CRP and serum amyloid P component. PTX3 is abundantly produced by various cells in atherosclerotic lesions, including monocytes, macrophages, endothelial cells, vascular smooth muscle cells, fibroblasts, dendritic cells, and adipocytes, whereas CRP is mainly produced in the liver [11]. These findings suggest that PTX3 levels reflect local inflammation at atherosclerotic lesions more accurately than does CRP.

In this study, we analyzed the relationship between carotid plaque vulnerability and serum inflammatory biomarkers and determined the expression of these soluble factors in carotid plaques.

## Materials and Methods

### Subjects

Forty-one patients underwent carotid artery stenting (CAS) and 17 patients underwent carotid endarterectomy (CEA) for symptomatic or asymptomatic carotid stenosis at Mie University Hospital between September 2009 and March 2012 in this prospective study. Patients who met our criteria for CAS included those diagnosed with carotid lesions that were either symptomatic with >50% stenosis or were asymptomatic with >80% stenosis, as assessed by digital subtraction angiography performed as described by the North American Symptomatic Carotid Endarterectomy Trial [12]. All patients who underwent CAS had at least one coexisting condition on specific clinical criteria that potentially increased the risk posed by CEA, according to SAPPHIRE study [13]. And we had 20 control subjects who had chronic-stage cerebral infarction without severe carotid artery stenosis (mean age 74, 14 men and 6 women).

Carotid artery stenosis was considered symptomatic if the patient had a history of ipsilateral ischemic events attributed to the affected carotid artery within the previous 120 days before CAS and CEA, and asymptomatic if no ischemic event occurred during this period.

This study was approved by the Ethical Committee of Mie University. Written informed consent was obtained from all the patients.

### Magnetic Resonance Imaging Assessments

Three days before CAS, magnetic resonance (MR) examinations, including 3D-T1 gradient echo (GRE) carotid plaque imaging, were performed on all patients after diagnostic angiography. No ischemic events, such as transient ischemic attack or stroke, occurred between pre-procedural MR examinations and CAS. MR imaging was performed using a 3.0-T MR imaging system (Achieva Quasar Dual, Philips Medical Systems, Best, The Netherlands).

3D-T1 GRE carotid plaque imaging was performed in the coronal plane with null blood conditions (effective inversion time, 600 ms; TR/TE, 5.0/2.3 ms) and the water excitation technique to suppress fat signals. Other scanning parameters were as follows: FOV, 260 mm; voxel size, 0.68×0.68×1.00 mm; flip angle, 13°; partitions, 56 partitions covering 70 mm around the carotid bifurcation; and data acquisition time, 4 min 2 s.

MR images were reviewed by a neurointerventionalist blinded to the clinical data. Regions of interest were drawn manually on a workstation around the carotid plaque and the adjacent sternocleidomastoid muscle (SCM) with coronal 3D-T1TFE images that detected the largest carotid plaque segment. The signal intensity ratio (SIR) was defined as the signal intensity of the carotid plaque divided by the signal intensity of SCM, being >1.8 for vulnerable plaques and ≤1.8 for stable plaques, as previously described [14].

### Procedures

All patients received aspirin (100 mg/day) and clopidogrel sulfate (75 mg/day), ticlopidine hydrochloride (200 mg/day), or cilostazol (200 mg/day) as anti-platelet therapy for at least 2 weeks before CAS. All CAS procedures were performed under local anesthesia via the percutaneous transfemoral route by an experienced neurointerventional team. Systemic blood samples were obtained via the femoral sheath immediately after insertion. A heparin bolus of 100 U/kg was intravenously administered immediately after the introducer sheath was placed to increase the activated clotting time to a minimum of 300 s. Two different types of distal filter embolic protection devices were used: Angioguard XP (Cordis, Minneapolis, MN) and FilterWire EZ (Boston Scientific, Natic, MA). Before advancing the distal filter device, flow arrest was obtained by temporary balloon occlusion using the Optimo balloon guiding catheter (Tokai Medical Products, Aichi, Japan) in the common carotid artery and PercuSurge GuardWire (Medtronic AVE, Santa Rosa, CA) in the external carotid artery. Then, the local stagnant blood near the atherosclerotic plaque area was slowly aspirated from the guiding catheter and collected as the pre-procedural local blood sample. Under distal filter protection, pre-dilatation with an angioplasty balloon was performed as necessary; a self-expandable stent–Precise (Cordis, Minneapolis, MN) or Carotid Wallstent (Boston Scientific, Natic, MA)–was deployed; and post-dilatation was done as necessary. After these procedures, the local stagnant blood was aspirated with temporary balloon occlusion and collected as the post-procedural local sample. When aspiration was complete, the distal filter device was captured and removed.

Blood sampling of control patients were drawn by veinpuncture.

Blood samples were centrifuged at 3000 rpm for 15 min, and the supernatants were separated and stored at −80°C until analysis. Debris captured by the distal embolic protection devices was fixed in 10% paraformaldehyde, placed in gel, embedded in paraffin, and sliced into 2-µm-thick sections.

All patients treated by CEA underwent general anesthesia. Intraluminal shunt insertion was performed immediately after arterectomy before removing the atherosclerotic plaques to provide immediate reperfusion. Before skin closure, all patients underwent angiography in the operating room to confirm patency of the carotid arteries. Atherosclerotic plaque samples collected during CEA were fixed in 10% formalin, embedded in paraffin, and sliced into 5-µm-thick sections.

### Measurement of Blood Inflammatory and Anti-inflammatory Markers

The serum levels of the proinflammatory cytokines: IL-1β, IL-6, interferon gamma (IFNγ), tumor necrosis factor alpha (TNFα), and matrix metalloproteinase (MMP)-9; endothelial activation markers: E-selectin, intracellular adhesion molecule 1 (ICAM-1), and vascular cell adhesion molecule 1 (VCAM-1); and anti-inflammatory cytokines: IL-10 and adiponectin, were measured using a multiplex bead array system (Luminex; Millipore, Billerica, MA, USA). Luminex assays were performed using 96-well microplates according to the manufacturer’s protocol.

A commercially available enzyme-linked immunosorbent assay (ELISA) was used to measure the serum levels of inflammatory markers: hs-CRP (CircuLex, Nagano, Japan) and PTX3 (Aviscera Bioscience, Inc., CA, USA).

### Histological Analysis

The samples from the CAS-treated patients were histologically examined to characterize the contents of the debris captured by the distal filter device. Filters were gently rinsed with saline and immediately fixed in 10% formaldehyde. Subsequently, the captured debris was extracted from each filter and placed in agarose gel followed by paraffin embedding.

In the patients who underwent CEA, whole specimens were fixed in 10% formalin, sectioned in 3.0-mm transverse slices, decalcified, and embedded in paraffin. The paraffin-embedded specimens were sectioned at 5-µm thickness stained with hematoxylin and eosin (HE), and subjected to histopathological and immunohistochemical analyses.

The sections obtained from the CEA-treated patients were stained with mouse anti-IL-6 antibody (monoclonal, diluted 1∶200, LifeSpan Biosciences, Seattle, WA, USA); rabbit anti-E-selectin antibody (polyclonal, diluted 1∶100, Santa Cruz Biotechnology, Santa Cruz, CA, USA); and rabbit anti-PTX3 antibody (monoclonal, diluted 1∶100, Enzo Life Sciences, Farmingdale, NY, USA). Deparaffinized sections were incubated with 1% (v/v) H_2_O_2_ in methanol for 3 min to eliminate endogenous peroxidase activity. After blocking with 10% (v/v) normal serum, the sections were incubated for 1 h at room temperature with the primary antibodies. After three 5-min washes in phosphate-buffered saline, the sections were incubated with biotinylated secondary antibodies for 1 h and then with the avidin-biotinylated horseradish peroxidase complex (ABC Elite kit, Vector, Burlingame, CA, USA) for 30 min. Peroxidase labeling was visualized using 0.2% (v/v) 3,3′-diaminobenzidine as a chromogen. When using the antibodies, the sections were lightly counterstained with hematoxylin. Morphological characteristics of carotid plaque were established on hematoxylin staining according to the previous classification [15]. The quantification of IL-6, PTX3 and E-selectin positive cells was established from immunohistochemical sections. Quantitative evaluation of 10 optical fields was performed at x100 magnification. Results are expressed as the number of cells per 10 fields.

### Data Analysis

During CAS, intracarotid blood samples were slightly diluted in normal saline and iodinated contrast medium. Therefore, the level was normalized for albumin to correct for dilution. Data are reported as means ± IQR (interquartile range). The chi-square test and Wilcoxon’s rank test were used to perform intergroup comparisons about patient characteristics. Shapiro Wilk test was used to evaluate normality, and Kruskal Wallis test was used to evaluate continuous variables for data measured by Luminex and ELISA. The Wilcoxon’s rank test was used for pair comparisons of cell counts about histological analysis. Receiver operating curve was constructed to identify the cutoff point of PTX3. The SPSS Statistics 22 software package was used to perform descriptive statistical analyses. P<0.05 was considered statistically significant.

## Results

### Patient Characteristics

The baseline characteristics of the patients with CAS are summarized in Table 1. Twenty patients showed SIR >1.8 and were classified into the vulnerable plaque group, while 21 had SIR ≤1.8 and were classified into the stable plaque group. All but 1 patient with vulnerable plaques were males. In terms of other demographic profiles, there were no differences between the 2 groups.

**Table 1:** Patient characteristics.

	Total	Stable n = 21	Vulnerable n = 20	p
Age, years, mean ± SD	64.2–79.5 (74.0)	73.0–79.0 (74.0)	69.3–79.8 (75.0)	NS
Gender, no (Male/Female)	34/7	15/6	19/1	<0.05
Body mass index, kg/m^2^	19.5–24.3 (21.0)	21.1–25.5 (22.7)	20.7–24.0 (21.9)	NS
eGFR, mL/min/1.73 m^2^	52.5–64.9 (59.2)	52.5–63.6 (57.6)	52.3–68.9 (61.5)	NS
Systolic blood pressure, mmHg	120.5–140.0 (132.0)	118.0–143.0 (129.0)	121.3–138.5 (132.5)	NS
Diastolic blood pressure, mmHg	66.0–83.0 (75.0)	62.5–79.0 (75.0)	69.3–88.8 (77.5)	NS
Carotid stenosis, %	50.0–95.0 (82.0)	71.5–90.0 (80.0)	50.0–95.0 (85.0)	NS
Symptomatic/Asymptomatic	30/11	17/4	13/7	NS
Previous disease				
Hypertension, no. (%)	35 (85.3)	19 (90.5)	16 (80.0)	NS
Diabetes mellitus, no. (%)	17 (41.5)	8 (38.1)	9 (45.0)	NS
Hyperlipidemia, no. (%)	23 (56.1)	13 (61.9)	10 (50.0)	NS
Ischemic heart disease, no. (%)	16 (39.0)	8 (38.1)	8 (40.0)	NS
Smoking, no. (%)	8 (19.5)	4 (19.1)	4 (20.0)	NS
History of chronic alcohol use, no. (%)	15 (36.6)	8 (38.1)	7 (35.0)	NS
Oral administration				
Aspirin, no. (%)	32 (78.5)	17 (81.0)	15 (75.0)	NS
Clopidogrel sulfate, no. (%)	22 (53.7)	13 (61.9)	9 (45.0)	NS
Ticlopidine hydrochloride, no. (%)	5 (12.2)	2 (9.5)	3 (15.0)	NS
Cilostazol, no. (%)	25 (61.0)	10 (47.6)	15 (75.0)	NS
ACE-I or ARB, no. (%)	28 (68.3)	14 (66.7)	14 (70.0)	NS
Statin, no. (%)	36 (87.8)	17 (81.0)	19 (95.0)	NS

eGFR: Estimated glomerular filtration rate; ACE-I angiotensin-converting enzyme inhibitor; ARB: angiotensin II receptor blocker.

### Serum Markers of Inflammation and Endothelial Activation

#### Investigation of serial changes in cytokines, endothelial activation, and inflammatory markers (Figure 1)

Several significant differences were observed in the IL-6, IL-10, E-selectin, VCAM-1, and PTX3 levels between local and systemic samples. Among proinflammatory markers, levels of IL-6 were higher in the post-procedural local samples than those in the systemic samples. The levels of the markers for endothelial activation–E-selectin and VCAM-1–and of the inflammatory marker PTX3 were also increased. For anti-inflammatory markers, IL-10 levels were higher in the post-procedural local samples than in the systemic samples. A comparison of serial changes in IL-6 and IL-10 levels showed significantly higher values in the post-procedural local samples than in the systemic samples. Further, E-selectin, VCAM-1 and PTX3 levels were significantly higher in the pre- and post-procedural local samples than in the systemic samples. No significant differences were observed in the IL-1β, TNFα, IFNγ, MMP-9, ICAM-1, adiponectin, and hs-CRP levels (Figure S1).

**Figure 1:**
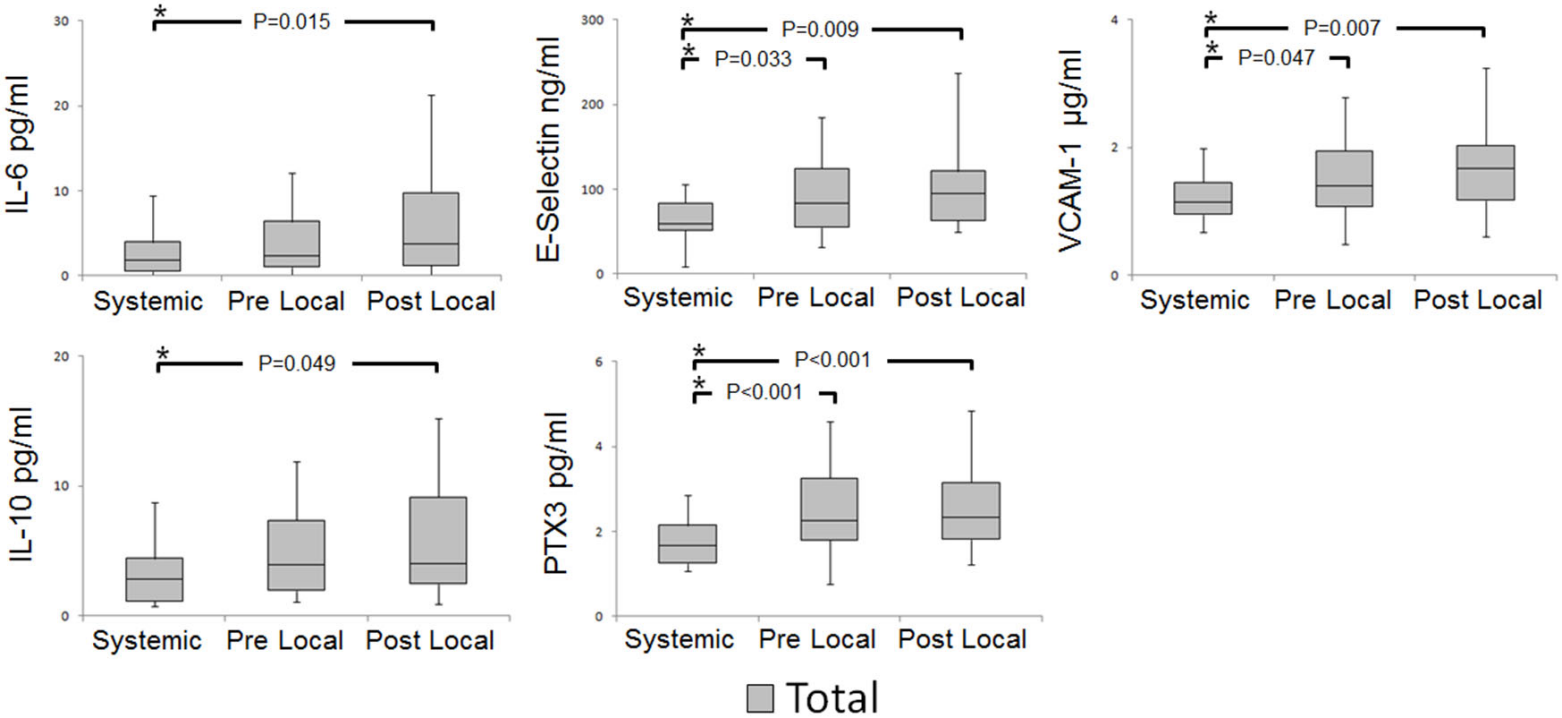
Systemic and local levels of proinflammatory and anti- inflammatory markers. Serum levels of inflammatory markers in systemic, pre-, and post-procedural local samples were measured by ELISA. Results are expressed as mean ± IQRs. Differences between the samples are not significant, except for those indicated by *P<0.05.

#### Comparison between stable and vulnerable plaques (Figure 2)

The levels of proinflammatory cytokines–IL-6 and TNFα; endothelial activation marker–E-selectin; and inflammation markers–hs-CRP and PTX3, were upregulated in the vulnerable plaque group as compared to the stable plaque group. In contrast, the levels of the anti-inflammatory cytokines IL-10 and adiponectin were downregulated in the vulnerable plaque group as compared to the stable plaque group. No significant differences were observed in the IFNγ, MMP-9, and ICAM-1 levels (Figure S2).

**Figure 2:**
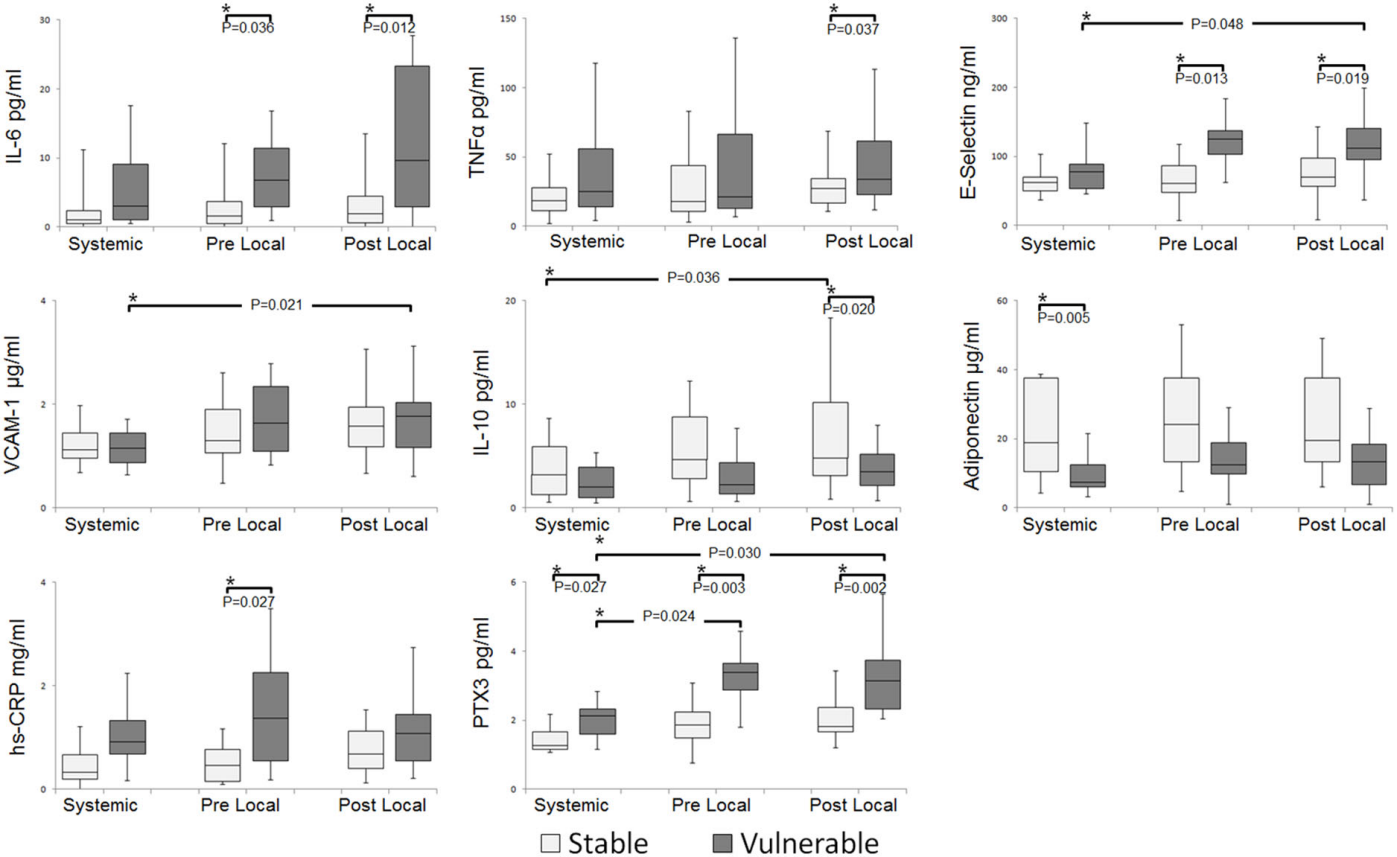
Comparison of proinflammatory and anti- inflammatory markers for plaque vulnerability. Serum levels of proinflammatory and anti-inflammatory markers from vulnerable and stable plaques in systemic, pre-, and post-procedural local samples were measured by ELISA. Results are expressed as mean ± IQRs. Differences between the samples are not significant, except for those indicated by *P<0.05.

IL-6 levels showed significant differences between the vulnerable and stable plaque groups in the post-procedural and pre-procedural local samples, and TNFα levels differed significantly in the post-local samples. E-selectin levels differed significantly between the systemic and post-procedural local samples in the vulnerable plaque group and between the vulnerable and stable plaque groups in the pre- and post-procedural local samples. Serial VCAM-1 levels also differed significantly between the systemic and post-procedural local samples. Significant differences were observed in the hs-CRP levels in the pre-procedural local samples between the vulnerable and stable plaque groups.

Among these markers, the most distinctive changes were observed in PTX3 levels, which were higher in the vulnerable plaque group than in the stable plaque group for systemic, pre-, and post-procedural local samples. Moreover, PTX3 levels differed significantly between the systemic, pre- and post-procedural local samples in the vulnerable plaque group.

The levels of anti-inflammatory markers IL-10 and adiponectin were higher in the stable plaque group than in the vulnerable plaque group. IL-10 levels differed significantly between the systemic and post-procedural local samples in the stable plaque group and between the vulnerable and stable plaque groups in the post-procedural local samples. The level of adiponectin in the stable group was significantly higher than in the vulnerable plaque group.

#### Comparison between patients with and without severe carotid artery stenosis (Figure 3)

Significant differences were observed in the adiponectin, hs-CRP, and PTX3 levels. PTX3 was upregulated in the vulnerable plaque group as compared to the stable plaque and control group, and hs-CRP was also upregulated in the vulnerable plaque group as compared to the control. For anti-inflammatory markers, the level of adiponectin was downregulated in the vulnerable plaque group as compared to the stable plaque group. No significant differences were observed in the IL-6, IL-1β, IL-10, TNFα, IFNγ, MMP-9, E-selectin, ICAM-1, and VCAM-1 levels (Figure S3).

**Figure 3:**
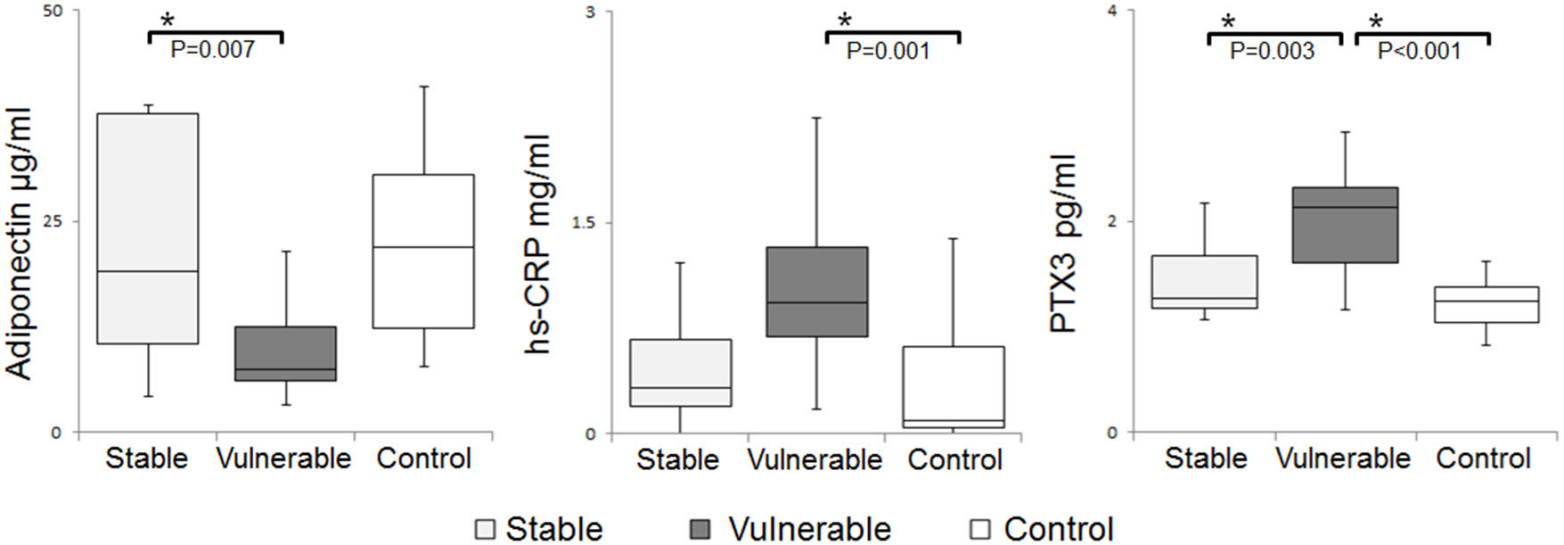
Comparison of systemic and control samples. Serum levels of proinflammatory and anti-inflammatory markers in systemic samples of patients with vulnerable and stable plaques, and controls were measured by ELISA. Results are expressed as mean ± IQRs. Differences between the samples are not significant, except for those indicated by *P<0.05.

### Histological Analysis of Captured Debris and CEA Samples (Figure 4)

To investigate the relationship between atherosclerosis and inflammation, we performed HE staining and immunohistochemical analyses. After histological analysis, the CEA plaques were classified as vulnerable (n = 12) or stable (n = 5). Vulnerable plaques from the CEA-treated patients showed hematomas, lipid-rich cores, and inflammatory cell infiltration, and those from CAS-treated patients showed inflammatory cell infiltration. The debris captured from the stable plaque group of CAS-treated patients showed cholesterin crystals.

**Figure 4:**
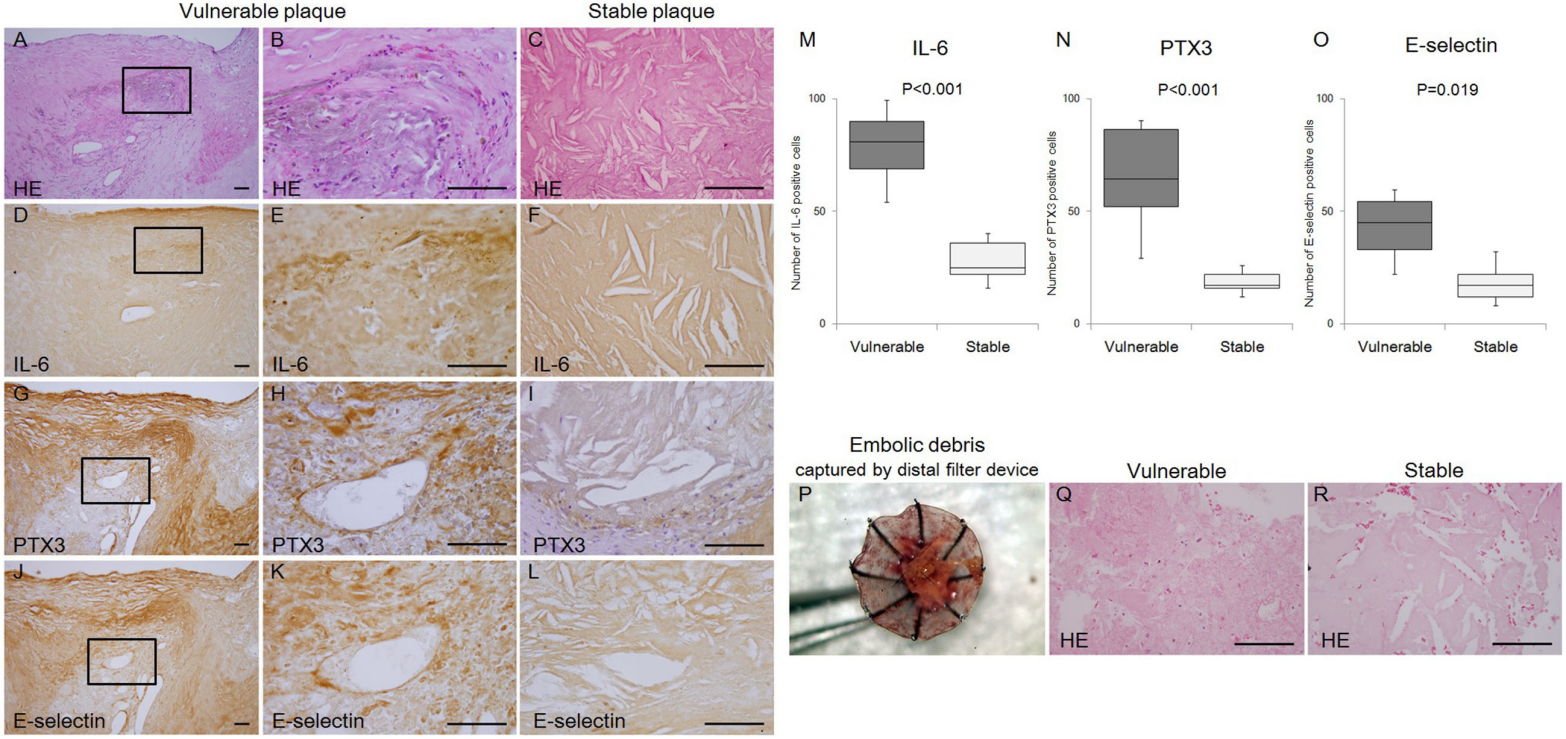
Immunochemical staining of atherosclerotic carotid endarterectomy specimens and emboli captured by distal protection devices. Serial sections were stained with hematoxylin and eosin (HE) (A, B, C, Q, R), anti-IL-6 (D, E, F), anti-PTX3 (G, H, I), and anti-E-selectin (J, K, L) antibodies. Sections from CEA (A to L), quantification of immunohistochemistry analysis of IL-6, PTX3, and E-selectin staining of sections from CEA (M, N, O), and embolic debris from CAS captured by the distal filter device (P, Q, R) are shown. HE staining showed inflammatory cell infiltrations in the vulnerable plaques (A, B, Q) and cholesterin crystals in the stable plaques (C, R). IL-6 staining is observed in the vulnerable plaques with inflammatory cell infiltration (D, E) but not in the stable plaques (F). PTX3 expression is observed in endothelial and perivascular cells and in the basement membrane in the vulnerable plaques (G, H) but not in the stable plaques (I). Immunohistochemistry revealed E-selectin expression in endothelial cells in the vulnerable plaques (J, K) but not in the stable plaques (L). The second line on the left of the vulnerable plaques (B, E, H, K) shows enlargement of the cropped areas. Scale bar, 100 µm.

All plaques from the CEA-treated patients showed IL-6, PTX3 and E-selectin expression. Quantitative analysis of IL-6-, PTX3- and E-selectin-positive cells was investigated. These three markers are more abundant in vulnerable plaques. IL-6 expression with inflammatory cell infiltration was observed in the vulnerable plaque group, while PTX3 expression was observed sporadically in endothelial and perivascular cells and basement membranes in the vulnerable plaque group of CEA-treated patients. E-selectin expression was observed in endothelial cells in the vulnerable plaque group of CEA-treated patients.

### Predictor of Vulnerable Plaque (Figure 5)

**Figure 5:**
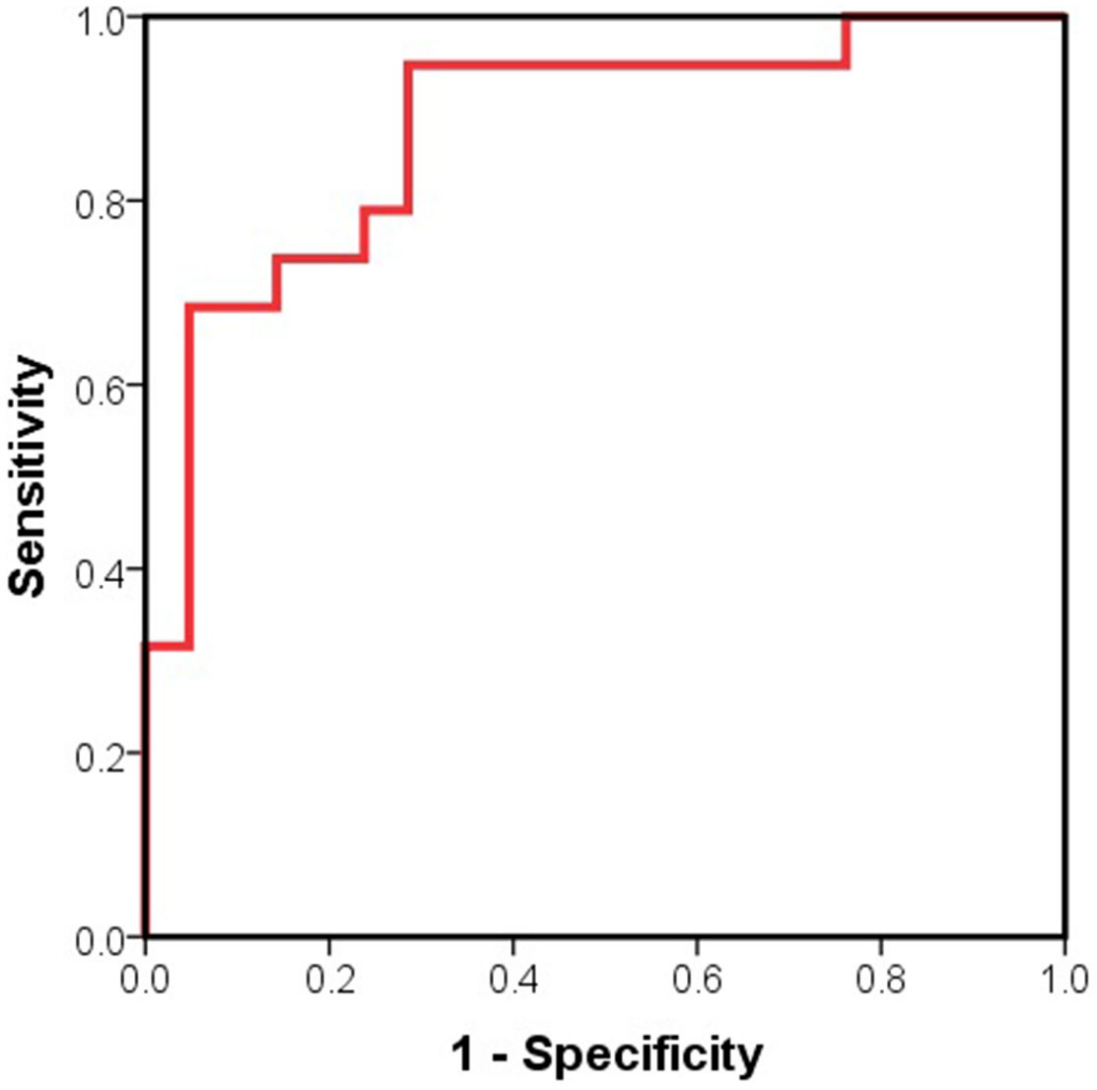
Receiver-operating curve of PTX3 levels for predicting vulnerable plaque. The area under the curve of PTX3 levels were obtained from all patients.

Figure 5 shows the receiver-operating curves of PTX3 levels for predicting vulnerable plaques. The area under the curve was 0.877 (95% confidence interval: 0.767 to 0.987; P<0.001). A PTX3 cutoff level of 1.55 pg/ml distinguished vulnerable plaques from stable plaques with 94.7% sensitivity and 71.4% specificity, and a PTX3 cutoff level of 1.93 pg/ml distinguished vulnerable plaques with 68.4% sensitivity and 95.2% specificity.

## Discussion

The present study revealed the following: (1) the vulnerability of carotid atherosclerosis was associated with certain soluble factors involved in inflammation, (2) PTX3 may become a potential new powerful predictor for determining plaque vulnerability, and (3) IL-10 and adiponectin were associated with plaque stability.

MR plaque imaging represents a new modality for risk assessment in atherosclerosis [16]–[18]. High-resolution MR imaging can detect the following characteristics of a vulnerable plaque: a thin or ruptured fibrous cap, a large lipid or necrotic core, and intraplaque hemorrhage [19]. A recent study reported high signal intensity (SIR >1.8) to be associated with cerebral embolism during CAS [14], and we accordingly categorized plaque vulnerability.

**Figure 6:**
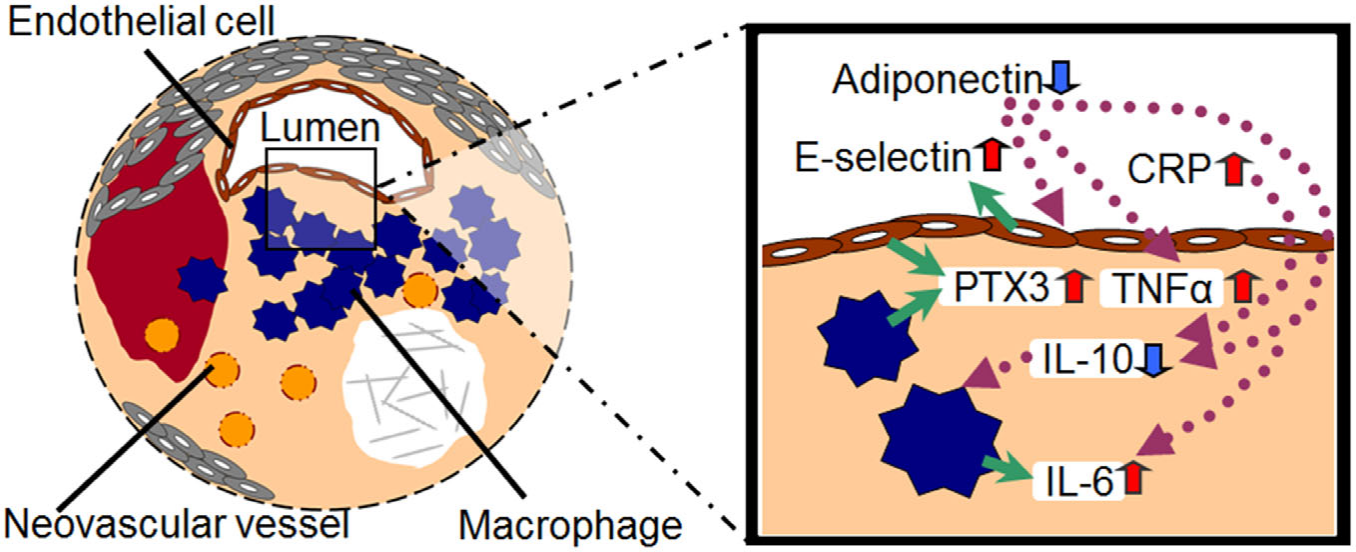
Proinflammatory and anti-inflammatory biomarkers in vulnerable plaques. Decreased adiponectin levels induce endothelial cell dysfunction with E-selectin release, with subsequent low IL-10 levels and high TNFα levels. Impaired endothelial cells release E-selectin, PTX3, and TNFα. Macrophages produce PTX3, TNFα, and IL-6.

Inflammatory mechanisms are known to play a central role in the pathogenesis and progression of atherosclerosis, plaque rupture, thrombosis, and stroke. Inflammatory biomarkers, such as hs-CRP, IL-6, E-selectin, ICAM-1, and VCAM-1, have been previously identified [20]–[23]. IL-6 has been reported to be higher in local samples from patients with carotid artery plaques, increasing further after CAS with periprocedural new ischemic lesions [24]. In present study, serum IL-6, VCAM-1, E-selectin and PTX3 levels were significantly higher in the post-procedural local samples as compared to the systemic samples. This suggests an association between carotid atherosclerosis and the local production of not only IL-6 but also E-selectin, VCAM-1 and PTX3 (Figure 6).

Plaque vulnerability is considered to be a major factor in stroke and should be accurately determined. The formation of carotid atherosclerotic plaques is a dynamic process in which modified lipids, extracellular matrix, monocyte-derived macrophages, and activated vascular smooth muscle cells accumulate in the arterial wall. In our study, the levels of several serum proinflammatory and anti-inflammatory markers, such as IL-6, IL-10, TNFα, E-selectin, adiponectin, hs-CRP, and PTX3, differed significantly between the vulnerable and stable plaque groups. Most of these markers occurred at higher levels in the vulnerable plaque group as compared to the stable plaque group, while IL-10 and adiponectin levels were decreased in the vulnerable plaque group. Previous reports have shown these markers to be associated with carotid atherosclerosis [24]–[26]. The present study further demonstrated that the upregulation of these proinflammatory markers and the downregulation of IL-10, an anti-inflammatory marker, were associated with plaque vulnerability. In particular, IL-6 expression was associated with inflammatory cell infiltration in the vulnerable plaques.

E-selectin was localized to the endothelial cells and inflammatory cells in the vulnerable plaques in tissue sections, as reported previously [27]. This finding suggested the occurrence of endothelial activation and a close relationship between plaque vulnerability and the production of these proinflammatory markers. In contrast, upregulated IL-10 and adiponectin expression in stable plaques may suggest a relationship between these cytokines and plaque stability. IL-10 is a potent anti-inflammatory cytokine with macrophage deactivating properties [28]. Adiponectin is an insulin-sensitizing protein expressed in adipose tissue that ostensibly plays a protective role in atherosclerosis and cardiovascular disease [29]. Low adiponectin levels have recently been associated with increased carotid intima-media thickness, which is indicative of atherosclerosis, being related to endothelial cell dysfunction and high E-selectin levels in patients with coronary heart diseases, whereas high adiponectin levels decrease the risk and play a protective role in atherosclerosis [30], [31]. Adiponectin induces IL-10 expression in leukocytes, which is correlated with decreasing IL-6 and TNFα levels [32], [33]. IL-10 inhibits macrophage activation; however, its production is decreased by CRP [34], [35]. Our results correspond with previous findings that have demonstrated an inverse association between adiponectin levels and atherosclerosis.

PTX3 has been suggested as a marker of inflammatory activity and plaque instability. Immunohistochemical studies have demonstrated increased PTX3 expression in atherosclerotic plaques as compared with that in non-atherosclerotic arteries [36], [37]. Matsuura et al have shown that PTX3 is more enhanced in vulnerable plaques than stable plaques in coronary artery of patients with angina pectoris and immunoreactivity for PTX3 is intense in the areas with intraplaque hemorrhage [38]. Previously, deficient symptomatology has indicated the absence of any relationship between PTX3 and the carotid artery [39]. In this study, serum PTX3 levels in both systemic and intracarotid samples before and after CAS were higher in the vulnerable group than in the stable group. And also, PTX3 expression was observed in inflammatory cells and in vascular endothelial and perivascular cells of vulnerable plaques. Therefore, our finding that PTX3 is associated with carotid plaque vulnerability is noteworthy. This finding corresponds to a previous report that suggested a potential association between PTX3 and stroke risk [40]. PTX3 is produced mostly in dendritic cells, endothelial cells, smooth muscle cells, macrophages, and fibroblasts [41]. Furthermore PTX3 may reflect the baseline atherosclerotic burden more accurately than CRP particularly under acute stress conditions, such as stroke, because of differences in amino acid sequences and molecular structures [40]. The close correlation observed between serum PTX3 levels and plaque vulnerability in the present study indicates that PTX3 may be a potential predictor of ischemic stroke.

Our present findings support the theory that PTX3 may be a potentially modifiable risk factor for atherosclerosis and that adiponectin may provide potentially substantial clinical benefits in the stabilization of carotid plaques.

## Conclusion

Carotid plaque vulnerability was modulated by the upregulation of proinflammatory factors and the downregulation of anti-inflammatory factors. In particular, PTX3 may be a potential novel indicator of plaque vulnerability.

## Supporting Information

Figure S1 **Systemic and local levels of proinflammatory and anti- inflammatory markers.** Serum levels of inflammatory markers in systemic, pre-, and post-procedural local samples were measured by ELISA. Results are expressed as mean ± IQRs. No significant differences were observed in the IL-1β, TNFα, IFNγ, MMP-9, ICAM-1, adiponectin, and hs-CRP levels.
(TIF)

Figure S2 **Comparison of proinflammatory and anti- inflammatory markers for plaque vulnerability.** Serum levels of proinflammatory and anti-inflammatory markers from vulnerable and stable plaques in systemic, pre-, and post-procedural local samples were measured by ELISA. Results are expressed as mean ± IQRs. No significant differences were observed in the IL-1β, IFNγ, MMP-9, and ICAM-1 levels.
(TIF)

Figure S3 **Comparison of systemic and control samples.** Serum levels of proinflammatory and anti-inflammatory markers in systemic samples of patients with vulnerable and stable plaques, and controls were measured by ELISA. Results are expressed as mean ± IQRs. No significant differences were observed in the IL-6, IL-1β, IL-10, TNFα, IFNγ, MMP-9, E-selectin, ICAM-1, and VCAM-1 levels.
(TIF)
