# Supplementary figures and images for: Inflammatory Biomarkers in Atherosclerosis: Pentraxin 3 Can Become a Novel Marker of Plaque Vulnerability
Source: PLoS One. 2014 Jun 17;9(6):e100045. doi: 10.1371/journal.pone.0100045 (PMC4061039; doi:10.1371/journal.pone.0100045)

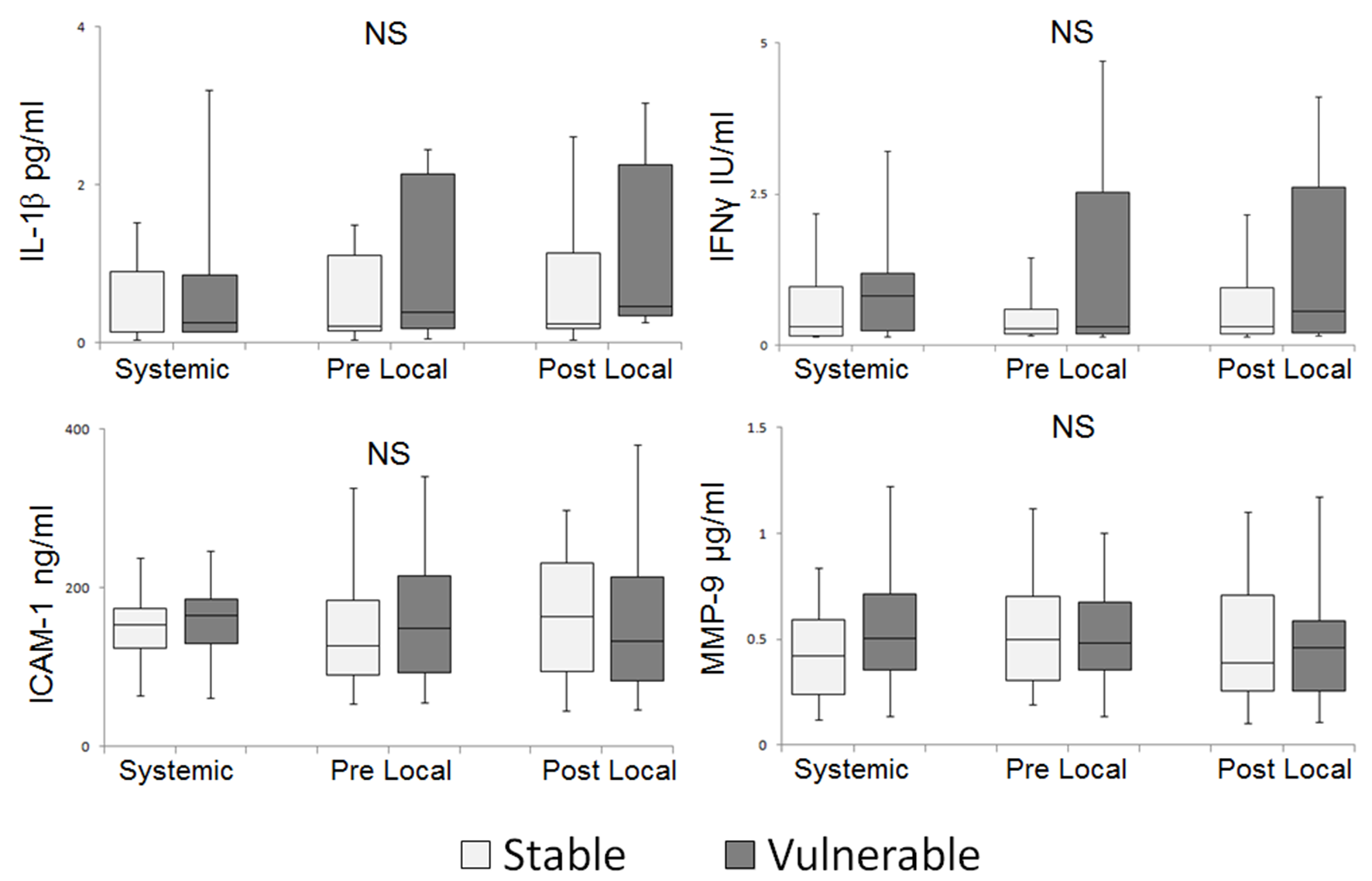

Supplement: Figure S2 — Comparison of proinflammatory and anti- inflammatory markers for plaque vulnerability. Serum levels of proinflammatory and anti-inflammatory markers from vulnerable and stable plaques in systemic, pre-, and post-procedural local samples were measured by ELISA. Results are expressed as mean ± IQRs. No significant differences were observed in the IL-1β, IFNγ, MMP-9, and ICAM-1 levels. (TIF) [file pone.0100045.s002.tif]

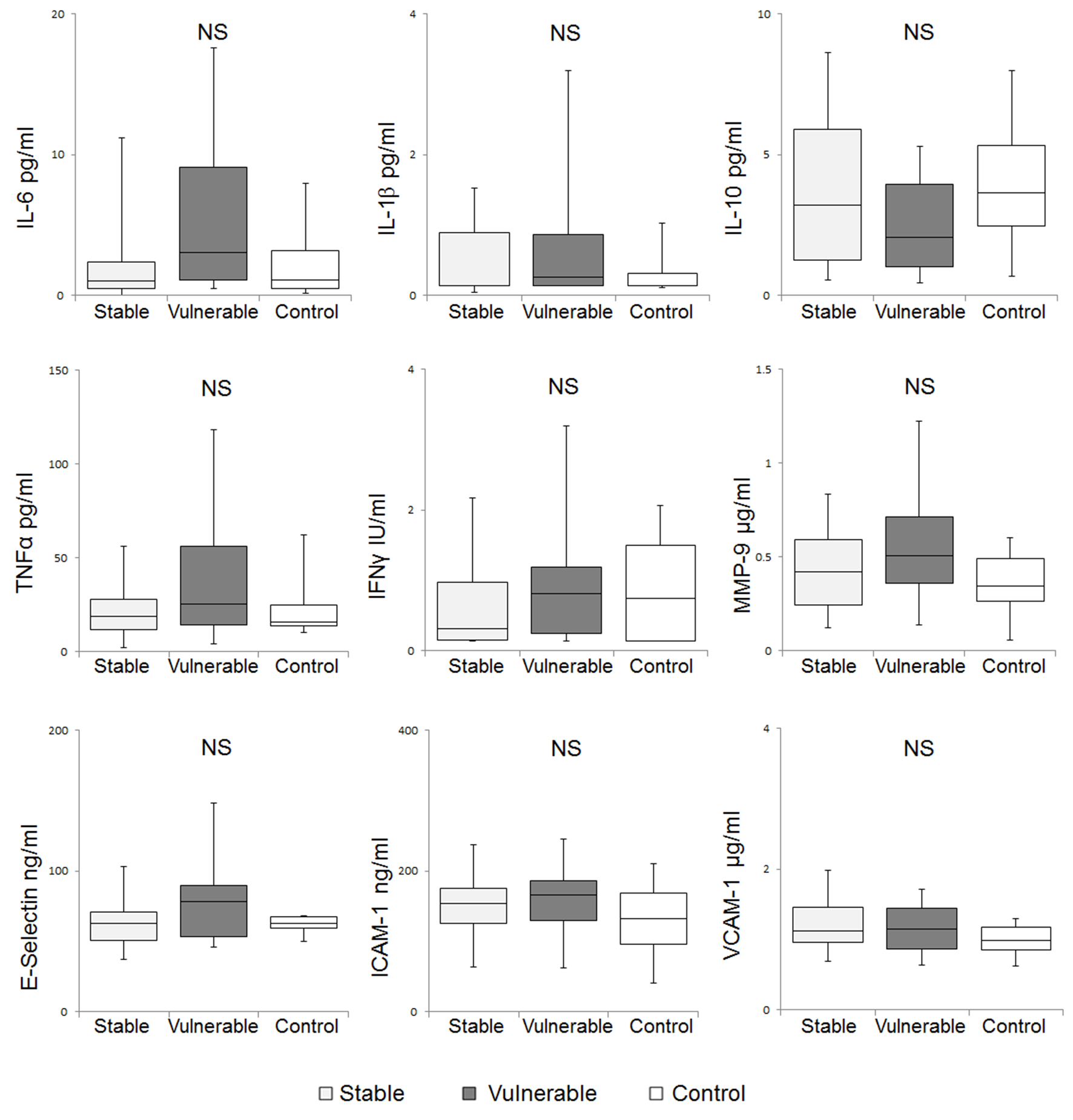

Supplement: Figure S3 — Comparison of systemic and control samples. Serum levels of proinflammatory and anti-inflammatory markers in systemic samples of patients with vulnerable and stable plaques, and controls were measured by ELISA. Results are expressed as mean ± IQRs. No significant differences were observed in the IL-6, IL-1β, IL-10, TNFα, IFNγ, MMP-9, E-selectin, ICAM-1, and VCAM-1 levels. (TIF) [file pone.0100045.s003.tif]
